# An unusual presentation of anomalous left coronary artery from the pulmonary artery (ALCAPA) syndrome in a 70-year-old man: a case report

**DOI:** 10.1186/s13256-018-1851-4

**Published:** 2018-10-22

**Authors:** Antonio Vizzuso, Riccardo Righi, Michela Zerbini, Stela Gamanji, Paolo Cucchi, Francesco Gallo, Melchiore Giganti, Giorgio Benea, Aldo Carnevale

**Affiliations:** 1 Department of Morphology, Surgery and Experimental Medicine, Section of Radiology, University of Ferrara, Via Ludovico Ariosto 35, 44121 Ferrara, Italy; 2 Department of Interventional and Diagnostic Radiology, Arcispedale Sant’Anna, Via Aldo Moro 8, 44124 Ferrara, Italy; 3 Department of Medical Science, Cardiovascular Institute, University of Ferrara, Via Ludovico Ariosto 35, 44121 Ferrara, Italy

**Keywords:** ALCAPA syndrome, Coronary vessel anomalies, Coronary angiography, Computed tomographic angiography, Coronary vessels

## Abstract

**Background:**

We present a rare case of anomalous origin of the left coronary artery from the pulmonary artery syndrome in an elderly man, and we describe coronary computed tomographic angiographic imaging findings to improve diagnostic confidence for the evaluation of this uncommon coronary artery anomaly.

**Case presentation:**

A 70-year-old Caucasian man came to our hospital with slight limitation of physical activity (New York Heart Association class II). He was asymptomatic for angina, syncope, and palpitations. Cardiac magnetic resonance imaging was performed after echocardiography because a hypertrophic cardiomyopathy was suspected; a plausible coronary artery anomaly was demonstrated as collateral evidence. Subsequently, coronary computed tomographic angiography showed the anomalous origin of left coronary artery from the pulmonary artery; the coronary vessels appeared markedly dilated and tortuous. Dilated intercoronary vessels along the epicardial surface of the heart and dilated bronchial arteries, corresponding to collateral pathways, were observed. Left ventricular hypertrophy, delayed subendocardial enhancement, and mitral insufficiency were better evaluated on magnetic resonance images. Invasive coronary angiography confirmed the main findings. Given the patient’s age and clinical performance, surveillance with medical management was considered appropriate, and surgical repair was avoided.

**Conclusions:**

Confidence with the anatomic pattern and clinical significance of this anomalous condition is necessary to improve cardiac imaging evaluation ability. In our patient, coronary computed tomographic angiography proved to be a reliable imaging approach, superior to invasive coronary angiography in terms of diagnostic performance and patient safety.

## Background

Anomalous left coronary artery from pulmonary artery (ALCAPA) syndrome (or Bland-White-Garland syndrome) is a rare, hemodynamically significant congenital coronary artery anomaly affecting 1 per 300,000 live births, accounting for 0.25–0.5% of all congenital cardiac diseases [1]. It is extremely rare for ALCAPA syndrome to be diagnosed in an adult, because the natural history of this malformation mostly leads to death within the first year of life if untreated [2]. The extent of collateral pathway development between the right and left coronary arteries (RCA and LCA, respectively) determines the extent of myocardial ischemia and the gravity of cardiovascular sequelae [3]. The aim of this article is to describe an unusual case of ALCAPA syndrome in a 70-year-old man with mild clinical manifestations.

## Case presentation

A 70-year-old retired Caucasian man, a former electrical society employee, presented to our hospital with moderate dyspnea on exertion (slight limitation of physical activity; New York Heart Association [NYHA] class II). He was asymptomatic for anginal pain, palpitations, or syncope. His past medical history included well-controlled hypertension, inguinal hernioplasty, and right total hip replacement for arthritis secondary to hip dysplasia. His family history revealed an unspecified heart condition in his father, who had died aged 55 years old. He denied smoking, drinking alcohol to excess, and use of recreational drugs. He did not have diabetes or hypercholesterolemia. He was receiving therapy with ramipril.

The patient’s vital signs at presentation were blood pressure 140/80 mmHg, pulse 58 beats/min with regular rhythm, and body temperature 36.0 °C. His body mass index was 30.8 kg/m^2^.

He appeared alert, oriented, and cooperative. His lungs were clear to auscultation and percussion bilaterally; no cardiac murmurs were appreciated. His abdomen was globose and nontender to palpation. His bowel sounds were normal in quality and intensity in all areas.

Mild lower extremity pitting edema was noted, but neither cyanosis nor clubbing was present.

Cranial nerves III–XII were intact; the results of motor and sensory examination of the patient’s upper and lower extremities were normal. The patient’s reflexes were normal and symmetrical bilaterally in both extremities.

The patient’s laboratory findings were unremarkable, except for a brain natriuretic peptide value of 1441 pg/ml (white blood cell [WBC] count 8.08 × 10^3^/μl, neutrophils 6 × 10^3^/μl, red blood cell [RBC] count 4.63 × 10^6^/μl, mean corpuscular volume 88 fl, mean corpuscular hemoglobin concentration 32 g/dl, hemoglobin 13.9 g/dl, platelets 218 × 10^3^/μl, creatinine 1.11 mg/dl, glomerular filtration rate 65 ml/min/m^2^, total protein 6.5 g/dl, urea 50 mg/dl, Na^+^ 142 mmol/L, K^+^ 3.8 mmol/L, Cl^−^ 98 mEq/L, glucose 77 mg/dl, bilirubin 0.58 mg/dl, alkaline phosphatase 67 U/L, alanine aminotransferase 19 U/L; normal urinalysis, with traces of RBCs, WBCs, and bacteria).

Transthoracic echocardiographic examination demonstrated a marked left ventricular concentric hypertrophy, with regular ventricular cavity size. The patient’s segmental/global kinetics were preserved, with borderline normal ejection fraction (55%); an expanded left atrium and a mild mitral regurgitation were noted.

**Fig. 1:**
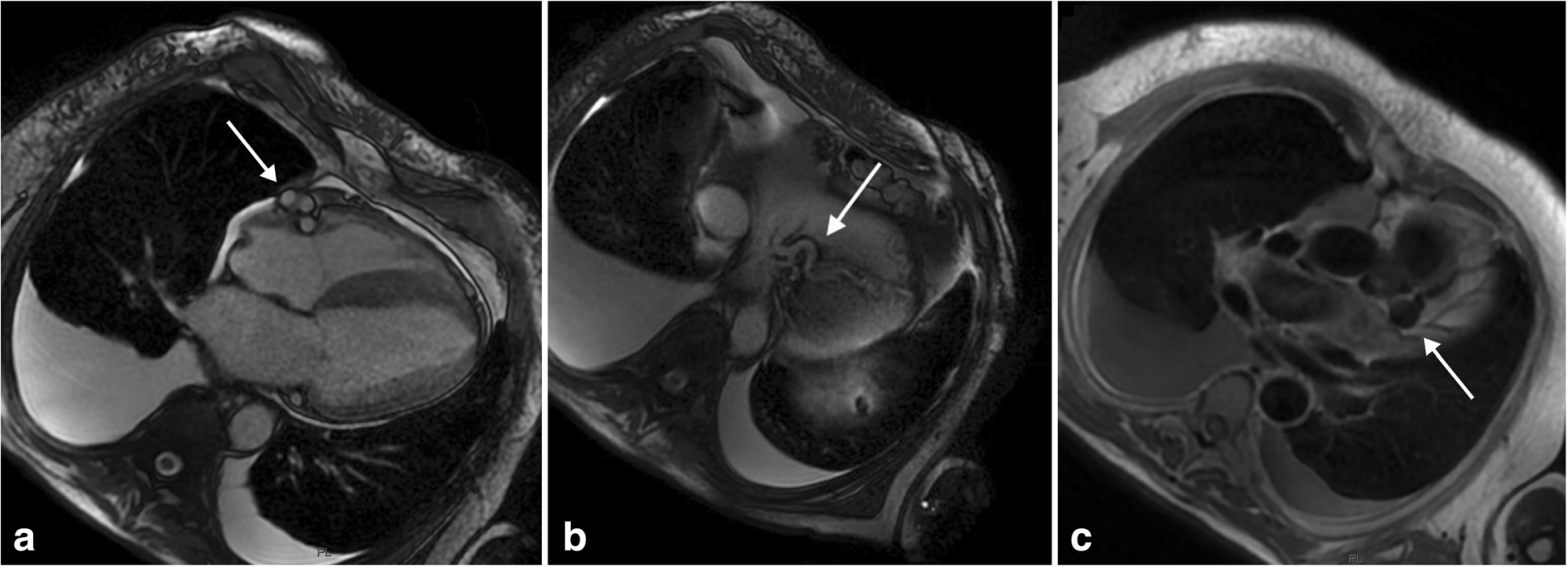
**a** and **b** Cardiac magnetic resonance imaging steady-state free precession cine sequence, four-chamber plane, showing a plausible coronary artery anomaly: dilated and tortuous right coronary artery (*arrow* in **a**), probably anastomosed with the distal portion of the left anterior descending artery (*arrow* in **b**). **c** T1-weighted black-blood sequence demonstrating a left coronary aneurysm (*arrow* in **c**), a feature suggestive of a coronary anomaly

**Fig. 2:**
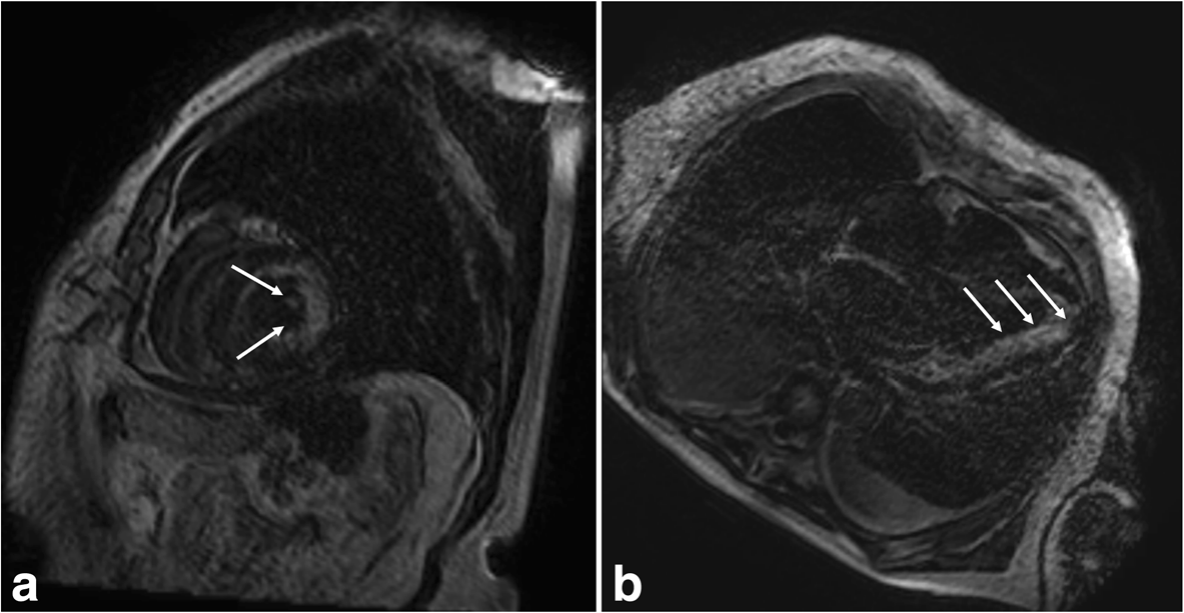
**a** and **b** Cardiac magnetic resonance imaging short-axis (**a**) and four-chamber (**b**) late gadolinium enhancement images showing diffuse subendocardial scar (*arrows*) at the lateral wall of the left ventricle, a finding consistent with subendocardial infarction subsequent to chronic subendocardial ischemia

Therefore, cardiac magnetic resonance (CMR) imaging was performed to investigate a suspected hypertrophic cardiomyopathy (Signa HDxt Echospeed 1.5-Tesla magnetic resonance imaging [MRI] scanner; GE Healthcare, Milwaukee, WI, USA). After administration of intravenous contrast material, late gadolinium enhancement (LGE) consistent with chronic subendocardial ischemia was revealed. As collateral evidence, CMR also showed a plausible coronary artery anomaly (Figs. 1 and 2).

Consequently, the patient underwent coronary computed tomographic angiography (CCTA) (Brilliance iCT 256-slice scanner; Philips Healthcare, Cleveland, OH, USA). A preliminary scan for scoring the amount of coronary calcium was obtained, and the Agatston score was calculated (0). Iodinated contrast media (Iopamiro 370; Bracco Imaging, Milan, Italy) was injected into an antecubital vein.

Nitroglycerin 0.3 mg was sublingually administered immediately before contrast injection. The patient was in sinus rhythm with a heart rate of 75–80 beats/min, unvaried after two 5-mg doses of intravenous metoprolol, so retrospective gated CCTA was performed, and reconstruction of cardiac phases from 40% to 78% of the R-R interval was done.

For proper scan timing, we used a bolus-tracking technique with an ROI placed in the ascending aorta. When the attenuation values in the selected area rose to a preset threshold (150 Hounsfield units), the system automatically played a short, prerecorded breath-hold instruction to the patient, and the scan was automatically commenced.

The following imaging parameters were used for data acquisition: 256 × 0.625 detector collimation, 270-ms gantry rotation time, 100-kV tube voltage and 618-mAs tube current-time product, and field of view of 18 cm with a matrix of 512 × 512. The dataset was reconstructed with a model-based iterative reconstruction, and the images were postprocessed and analyzed on an external workstation (Extended Brilliance Workspace; Philips Healthcare).

**Fig. 3:**
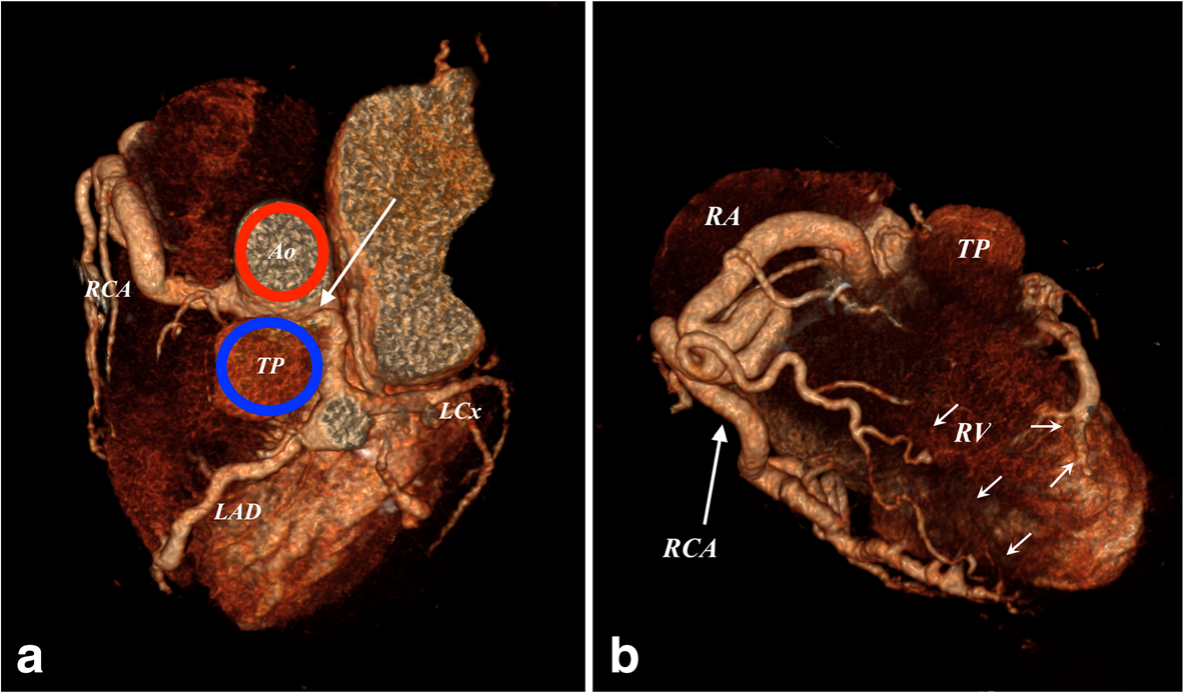
**a** and **b** Three-dimensional volume-rendered computed tomographic angiograms showing the anomalous origin of the left coronary artery from the pulmonary artery syndrome (*arrow* in **a**). Dilated intercoronary arteries (*short arrows* in **b**) can be seen along the epicardial surface, representing collateral pathways between the RCA and the left coronary artery. *Ao* Aorta, *LAD* Left coronary artery, *LCx* Left circumflex coronary artery, *RA* Right atrium, *RCA* Right coronary artery, *RV* Right ventricle, *TP* Pulmonary trunk

**Fig. 4:**
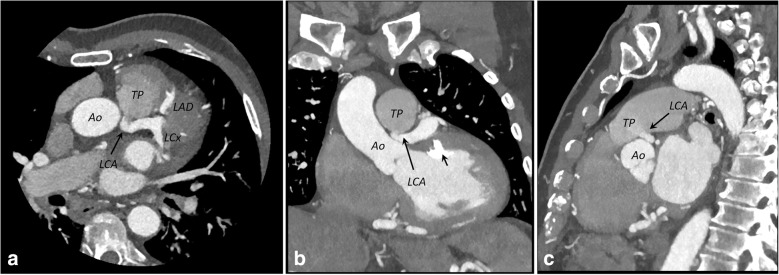
**a**–**c** Axial (**a**), coronal (**b**), and sagittal (**c**) maximum-intensity projection (5 mm) coronary computed tomographic angiographic images depicting the origin of the left coronary artery (LCA) (*arrows*) from the pulmonary trunk (TP) and not from the Aorta (Ao). Several calcifications are noted at the papillary muscle (*short arrow* in **b**), resulting from chronic ischemic changes. *LAD* Left coronary artery, *LCx* Left circumflex coronary artery

**Fig. 5:**
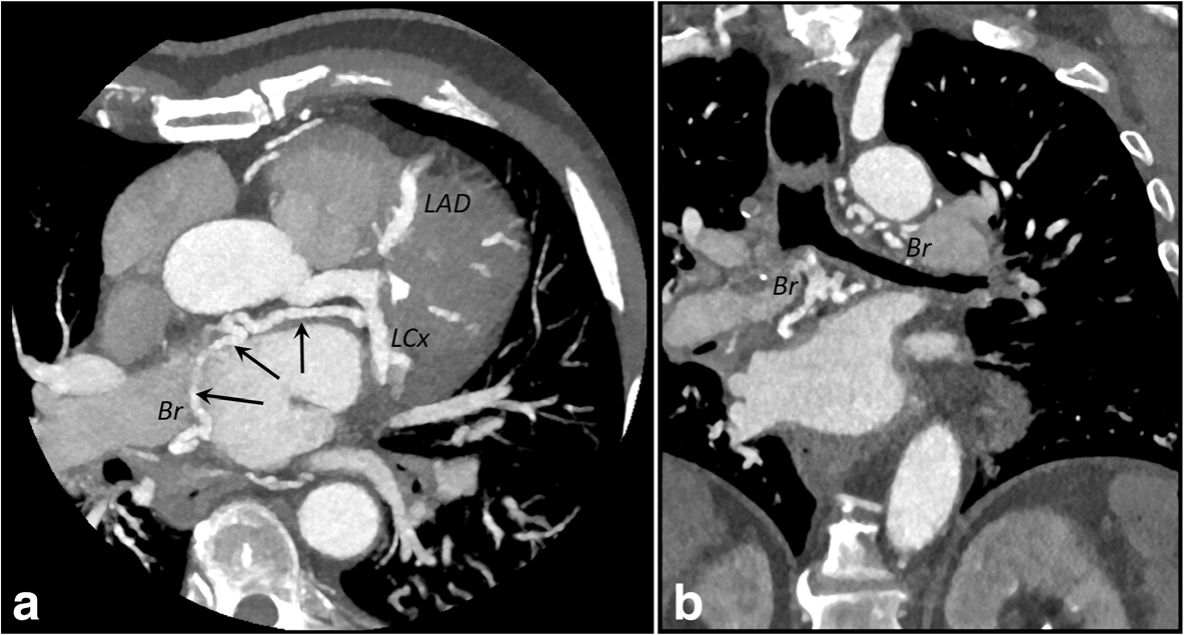
**a** and **b** Axial (**a**) and coronal (**b**) maximum-intensity projection (10 mm) coronary computed tomographic angiographic images showing dilated bronchial arteries (Br) anastomosing to the left circumflex artery (LCx) (*arrows*). *LAD* Left coronary artery

CCTA imaging clearly revealed that the LCA originated from the pulmonary artery and then bifurcated into the left anterior descending artery and the left circumflex artery. The RCA arose from the right coronary sinus, as normal. The RCA and LCA appeared markedly dilated and tortuous (Figs. 3 and 4). Dilated intercoronary vessels along the epicardial surface of the heart and dilated bronchial arteries corresponded to the collateral pathways of the LCA with the RCA and with systemic vessels, respectively (Fig. 5). The retrograde flow from the anomalous coronary artery to the main pulmonary artery was well depicted (steal phenomenon). The coronary arteries were smooth with no evidence of atherosclerotic disease. Several calcifications at the papillary muscle were observed.

**Fig. 6:**
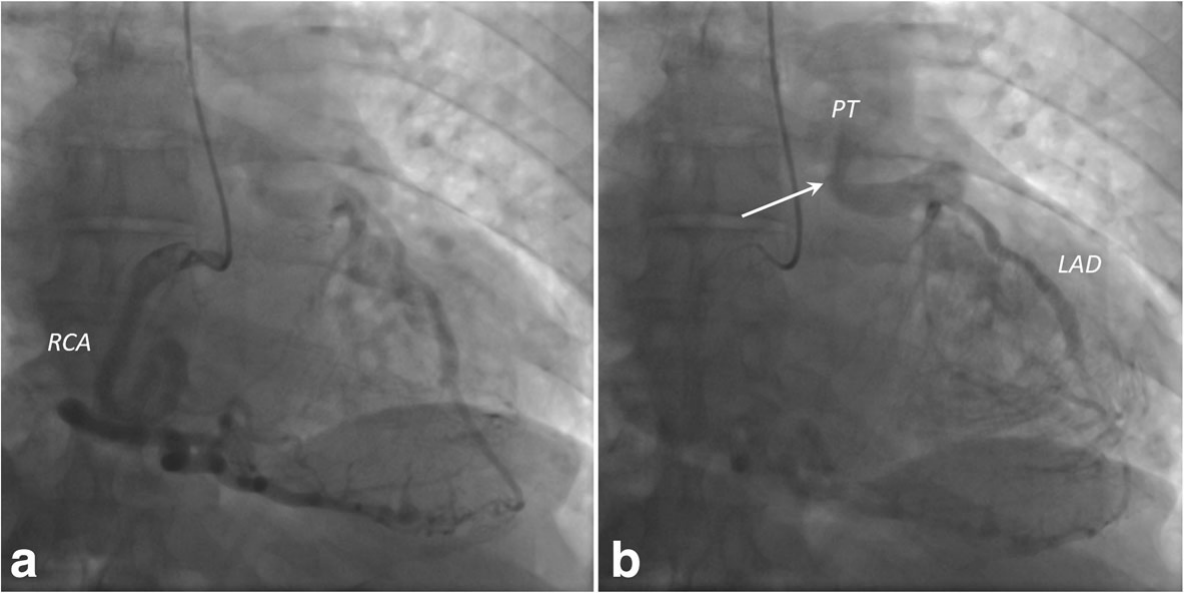
**a** Coronary angiogram obtained after right coronary artery (RCA) injection showing tortuous and dilated lumen. **b** Coronary angiogram demonstrating opacification of both the left anterior descending artery (LAD) and the pulmonary trunk (PT). This finding represents the “steal phenomenon” (*arrow*), the main diagnostic feature of left coronary artery from the pulmonary artery syndrome

After CCTA, invasive coronary angiography (ICA) was performed. First access was made through the radial artery, then another access was attempted through the femoral vein to confirm the origin of the LCA from the pulmonary artery (Fig. 6).

The risk/benefit ratio for surgery or surveillance with medical treatment was carefully analyzed; owing to the patient’s age, signs of mild chronic myocardial ischemia, and only slight limitation of physical activity, a therapy for ischemic cardiomyopathy (including bisoprolol 1.25 mg/d, furosemide 25 mg/d, canrenone 25 mg/d, ramipril 1.25 mg/d, apixaban 5 mg twice daily) was administered, and periodic surveillance was started. In our center, we do not have a standardized follow-up protocol for adult patients with ALCAPA syndrome. In this specific case, our patient underwent a cardiological visit, based on clinical examination and transthoracic echocardiography, 6 months after ICA. The patient was still symptomatic for dyspnea caused by slight physical activity (NYHA class II); an echocardiogram demonstrated stable chamber measures and ejection fraction (left ventricular ejection fraction, 55%), and mitral regurgitation was still moderate. The estimated pulmonary artery pressure was 55 mmHg.

## Discussion

This case report describes a 70-year-old man who presented to our hospital with slight limitation during ordinary activity, and he was diagnosed with ALCAPA syndrome. Owing to the rarity of this condition and its atypical presentation in adulthood, diagnosis can be challenging. Until recently, conventional coronary angiography was the method of choice for detecting coronary anomalies. We present our experience with the diagnosis of ALCAPA syndrome performed by CCTA, highlighting the outstanding features of this disease.

An anomalous origin of a coronary artery from the pulmonary artery is a rare condition, and ALCAPA syndrome represents the most common variant. Other types include anomalous origin of the RCA from the pulmonary artery and anomalous left anterior descending coronary artery or circumflex artery from the pulmonary artery [4]. It is a very rare situation, indeed, when ALCAPA syndrome is diagnosed in an adult, because the natural history of this malformation mostly leads to death within the first year of life if untreated [2].

Two types of ALCAPA syndrome are recognized: an infantile variant and a rarer adult variant [5, 6]. This anomaly has generally no effect on the hemodynamics of the coronary arteries in the prenatal and early neonatal phases, because blood pressure conditions between the systemic circulation and pulmonary arterial circulation are equalized by the patent ductus arteriosus, thus ensuring an antegrade blood flow in the LCA. After birth, the physiological occlusion of the ductus arteriosus lowers the pressure in the pulmonary arterial circulation, causing a decreasing flow rate in the LCA and a conversion of blood flow in the LCA toward the pulmonary artery. In the infantile variant, collaterals do not form between the RCA and LCA, and the retrograde flow in the LCA to the pulmonary artery results in steal of blood from the myocardium, with consequent myocardial ischemia, congestive heart failure, and mitral insufficiency. In infants, ALCAPA syndrome typically manifests with a failure to thrive, profuse sweating, pallor, dyspnea, and atypical chest pain while eating or crying.

The adult type is characterized by the compensatory formation of collaterals between the RCA and the LCA [1]. The extent of collateral circulation between the vessels during the critical period, when pulmonary arterial pressure gradually decreases, determines the extent of myocardial ischemia. The spectrum of clinical manifestations ranges from an asymptomatic course to decompensation of the coronary arterial collateral circulation, with the result of clinical evidence of a previously chronically subclinical ischemia. Ischemic cardiomyopathy, mitral insufficiency, or malignant dysrhythmias causing sudden death may occur secondary to myocardial ischemia [3, 5].

Echocardiography aids in diagnosis, notably in pediatric patients and young adults, because it allows the direct visualization of the abnormal origin of the LCA, dilated RCA, retrograde filling of the anomalous coronary artery, abnormal diastolic flow in the pulmonary artery, and abnormal septal or epicardial color flow signals from the collateral vessels [6, 7]. In our patient, the quality of the echocardiographic images was suboptimal owing to overweight and poor acoustic window: Neither the anomalous ostium of the LCA nor the course of the RCA was noticeable.

In recent years, CCTA has emerged as the standard of reference for identification and characterization of coronary artery anomalies. CCTA indeed provides a noninvasive imaging tool to demonstrate the origin and relationship of anomalous arteries to other mediastinal vascular structures, and it enables the use of three-dimensional reformation for delineation of subtle variations in the position and morphology of anomalous vessels [1]; hence, it could be considered the imaging modality of choice to noninvasively delineate coronary vessel anatomy. Moreover, it plays an important role in surgical intervention planning, and it may be a valuable postoperative follow-up tool for adult patients [8]. The short examination time and minimal aftercare make CCTA more practical than ICA [9]. Furthermore, advanced techniques and acquisition protocols, associated with improvements in the reconstruction phase, have enabled progressive dose decrease [10].

CMR imaging is a valuable, noninvasive imaging technique with the ability to assess myocardial viability and function. It can also be used to demonstrate the coronary arteries; however, long examination time and low spatial resolution are the main disadvantages compared with CCTA, limiting its application to demonstrating coronary arteries and their abnormalities [9].

MRI has an advantage over computed tomography (CT) in that it enables concurrent assessment of left ventricular and mitral valve dysfunction as well as signs of chronic myocardial ischemia (namely wall motion abnormalities and LGE) without the use of ionizing radiation. It can also directly demonstrate the reversed flow from the anomalous coronary artery into the pulmonary artery using steady-state free precession cine MRI, and it can help to quantify the degree of left-to-right shunting with phase-contrast imaging. Finally, MRI stress-rest perfusion and LGE imaging techniques can help identify reversible ischemia and subendocardial scarring [3–5]. Delayed subendocardial enhancement seen on MRI scans, a finding caused by subendocardial infarction resulting from chronic subendocardial ischemia, is relevant, especially in asymptomatic adults and those in whom ALCAPA syndrome is incidentally discovered, because it may be predictive of the onset of malignant dysrhythmias. If subendocardial enhancement is seen, surgical repair should be considered [3].

On CCTA imaging, direct visualization of the LCA arising from the main pulmonary artery is the diagnostic hallmark of ALCAPA syndrome. The LCA typically originates from the left inferolateral aspect of the main pulmonary artery just beyond the pulmonary valve. Both the RCA and LCA appear dilated and tortuous in adulthood, with a characteristic “steal phenomenon” (steal of blood flow from the myocardium) well depicted on MRI/CT scans as a retrograde flow from the LCA to the main pulmonary artery. Dilated intercoronary collateral arteries along the epicardial surface of the heart or within the interventricular septum and dilated bronchial arteries can also be observed, representing the collateral pathways between the RCA and the LCA and the systemic collateral vessels to the LCA. Left ventricular hypertrophy and dilation, left ventricular or most commonly global hypokinesis, subendocardial LGE on MRI studies, mitral insufficiency and prolapse, or several calcifications are findings of chronic ischemic changes [1, 3, 4, 9].

The main differential diagnosis for dilation of the coronary arteries includes vasculitis, such as Kawasaki disease or Takayasu arteritis, coronary artery-coronary sinus fistula, atresia, and atherosclerosis-related coronary artery ectasia [3]. Also, ALCAPA syndrome is among the differential diagnoses for massive cardiomegaly in the newborn period.

Surgical repair is the definitive treatment for ALCAPA syndrome, with the aim being to restore a two-coronary artery circulation system. Surgery is performed to correct subendocardial ischemia, thus improving left ventricular function and reducing the risk of malignant arrhythmia and sudden death [4]. Surgery is beneficial for pediatric patients with ALCAPA syndrome because it improves the survival rate; when contemplated for an elderly patient, one must consider unnecessary surgical risk without any clear improvement in the clinical outcome. It may be considered more prudent to medically treat these patients [6], as in our patient’s case.

Several surgical approaches are described in the literature. Coronary button transfer (or coronary arterial translocation) is the preferred surgical reparation technique for ALCAPA syndrome, especially in neonates, with low mortality rates and an excellent long-term prognosis. In this strategy, the proximal tract of the anomalous coronary artery is mobilized and anastomosed to the aortic root, whereas the pulmonary trunk is repaired with autologous pericardium. Other operative strategies include the Takeuchi repair (the creation of an aortopulmonary window and an intrapulmonary tunnel that baffles the aorta to the ostium of the anomalous LCA) and bypass graft to restore a two-coronary artery system with ligation of proximal anomalous artery [4]. Long-term follow-up imaging is recommended to evaluate for postsurgical complications.

## Conclusions

Coronary artery anomalies include a wide spectrum of variants with diverse cardiovascular manifestations. Familiarity with the anatomic pattern and clinical significance of each anomaly is necessary to categorize each condition and understand its importance in terms of prognosis. With the increasing use of modern imaging techniques such as CCTA and MRI, many asymptomatic patients are now diagnosed incidentally, as in our patient’s case. In particular, ALCAPA syndrome is a rare condition, even more unusual if encountered in an elderly patient.

## Abbreviations

ALCAPA: Anomalous left coronary artery from pulmonary artery
CCTA: Coronary computed tomographic angiography
CMR: Cardiac magnetic resonance
CT: Computed tomography
ICA: Invasive coronary angiography
LCA: Left coronary artery
LGE: Late gadolinium enhancement
MRI: Magnetic resonance imaging
NYHA: New York Heart Association
RBC: Red blood cell
RCA: Right coronary artery
WBC: White blood cell
